# Cone Synaptic Function is Modulated by the Leucine-Rich Repeat Adhesion Molecule LRFN2

**DOI:** 10.1523/ENEURO.0120-23.2024

**Published:** 2024-03-12

**Authors:** Nazarul Hasan, Ronald G. Gregg

**Affiliations:** ^1^Departments of Biochemistry & Molecular Genetics, University of Louisville, Louisville, Kentucky 40202; ^2^Ophthalmology & Visual Sciences, University of Louisville, Louisville, Kentucky 40202

**Keywords:** cone synapses, electroretinogram, LRFN2, LRFN2 knock-out mice

## Abstract

Daylight vision is mediated by cone photoreceptors in vertebrates, which synapse with bipolar cells (BCs) and horizontal (HCs) cells. This cone synapse is functionally and anatomically complex, connecting to eight types of depolarizing BCs (DBCs) and five types of hyperpolarizing BCs (HBCs) in mice. The dendrites of DBCs and HCs cells make invaginating ribbon synapses with the cone axon terminal, while HBCs form flat synapses with the cone pedicles. The molecular architecture that underpins this organization is relatively poorly understood. To identify new proteins involved in synapse formation and function we used an unbiased proteomic approach and identified LRFN2 (leucine-rich repeat and fibronectin III domain-containing 2) as a component of the DBC signaling complex. LRFN2 is selectively expressed at cone terminals and colocalizes with PNA, and other DBC signalplex members. In LRFN2 deficient mice, the synaptic markers: LRIT3, ELFN2, mGluR6, TRPM1 and GPR179 are properly localized. Similarly, LRFN2 expression and localization is not dependent on these synaptic proteins. In the absence of LRFN2 the cone-mediated photopic electroretinogram b-wave amplitude is reduced at the brightest flash intensities. These data demonstrate that LRFN2 absence compromises normal synaptic transmission between cones and cone DBCs.

## Significance Statement

Signaling between cone photoreceptors and the downstream bipolar cells is critical to normal vision. Cones synapse with 13 different types of bipolar cells forming an invaginating ribbon synapses with eight types, and flat synapses with five types, to form one of the most complex synapses in the brain. In this report, a new protein, LRFN2 (leucine-rich repeat and fibronectin III domain-containing 2), was identified that is expressed at the cone synapse. Using *Lrfn2* knock-out mice we show LRFN2 is required for the normal cone signaling.

## Introduction

Vision begins with the absorption of photons by opsins in rod and cone photoreceptor (PR) cells, which results in a glutamatergic signal at the PR axon terminal. This signal is relayed to postsynaptic bipolar and horizontal cells at the first synapse. The majority of the photoreceptors are rods, which function under dim light conditions. Rod-mediated vision has high sensitivity but relatively low acuity in humans. Rods make synapses primarily with a single type of depolarizing bipolar cell (DBC) and horizontal cells (HCs). Cones function under bright light conditions, have lower sensitivity than rods, and in humans mediate our high acuity vision.

Cones connect to eight types of DBCs and five types of hyperpolarizing bipolar cells (HBCs) in mice (West and Cepko, 2022). Cone DBCs, along with HCs dendrites, make invaginating synapses with cone axon terminals and HBCs make flat contacts with the cone pedicle base. The bipolar cells make connections with about forty types of retinal ganglion cells (RGC) (Bae et al., 2018), which transmit the signal to the rest of the brain. DBCs use the metabotropic glutamate receptor type 6 (mGluR6) (Nakajima et al., 1993) to detect glutamate concentration in the synapse and modulate membrane potential via the TRPM1 channel (Bellone et al., 2008; Morgans et al., 2009; Shen et al., 2009; Koike et al., 2010). In contrast, HBCs signal through AMPA/kainate-type iGluR receptors (Borghuis et al., 2014; Ichinose and Hellmer, 2016). BCs also can be distinguished based on cellular morphology (Kim et al., 2014; Greene et al., 2016) and gene expression pattern (Shekhar et al., 2016).

In rod BCs and cone DBCs, a signalplex that detects glutamate changes includes the mGluR6 receptor, GPR179 and TRPM1, as well as several associated partners (R9AP, RGS7/11, Gβ5). The correct assembly of the signalplex requires several known trans-synaptic organizing proteins that contain leucine-rich repeats (LRRs), including nyctalopin, ELFN1, ELFN2, LRIT3, and the dystroglycan complex. Nyctalopin is required for insertion of TRPM1 into the complex (Pearring et al., 2011). ELFN1 and 2 are required for insertion of mGluR6 in synapses involving rod and cone DBCs, respectively (Cao et al., 2020, 2022). The loss of LRIT3, results in the loss of nyctalopin and TRPM1 from the rod bipolar signalplex (Neuille et al., 2015; Hasan et al., 2019), and all the key signalplex proteins, including mGluR6, GPR179, nyctalopin, TRPM1, RGS7/11, and R9AP in cone DBCs. This notable difference between the impacts of loss of LRIT3 on rod BC vs cone DBC signalplexes led to our hypothesis that additional unknown synaptic protein(s) are likely required for rod and/or cone DBC signalplex assembly.

In this study, we used a proteomic approach to identify an additional synaptic cell adhesion molecules, LRFN2 (leucine-rich repeat and fibronectin III domain-containing 2) protein in mouse retina. LRFN2, also known as SALM1 (synaptic adhesion-like molecule-1), is a type I transmembrane glycoprotein with the N-terminal region that contains LRRs, immunoglobulin-like (Ig), and fibronectin type III (Fn3) domains, located in the extracellular space, and the intracellular C-terminal contains a PDZ binding domain (Morimura et al., 2006). Here, we show that LRFN2 is localized to the cone terminals. The knock-out of LRFN2 does not alter expression of TRPM1 or several other signalplex proteins, mGluR6, GPR179, ELFN2, and LRIT3; and conversely they are not needed for normal expression of LRFN2. The lack of LRFN2 has no impact on the scotopic electroretinogram but does result in a decrease in the photopic b-wave amplitude at the highest flash intensities. This adds another member of a growing group of synaptic proteins that interact with and/or organize the postsynaptic signaling complex in DBCs.

## Materials and Methods

### Animals

All procedures were performed in accordance with local Institutional Animal Care and Use Committees and the Society for Neuroscience policies for the use of animals in research. All mice were multiply housed in a local AAALAC (Association for Assessment and Accreditation of Laboratory Animal Care)-approved facility under a 12 hr light/dark cycle. All mouse lines used have been described previously: *Lrit3^−/−^* (Hasan et al., 2019)*, Trmp1^−/−^* (Shen et al., 2009), *Grm6^−/−^* (Masu et al., 1995), and *Gpr179^−/−^* (Peachey et al., 2012). These lines were either generated on a C57BL/6J background or backcrossed onto this background for at least ten generations. Mice between the ages of P45 and P100 of either sex were used in the experiments.

### Generation of *Lrfn2^−/−^* and *Elfn2^−/−^* mice with zinc finger nucleases

To create a null allele of *Lrfn2* we contracted with Sigma to develop a ZFN (zinc finger nuclease) targeted to a highly conserved region in exon 2 of *Lrfn2*. mRNA (10 ng/µl) encoding the ZFNs were injected into 350 C3H/HeNTac/C57BL/6NTac hybrid embryos. 250 viable embryos were implanted into eight Swiss Webster recipient mothers, yielding many offspring. Tail biopsies from offspring were collected, and genomic DNA isolated by digesting tissue in Direct PCR solution (Thermo Scientific) supplemented with 2.5 μg/μl proteinase K (Thermo Scientific). Primers flanking the ZFN target site (Fwd: 5′-TAACCTGGGCATAGCCTGTC-3′; Rev: 5′-AAGGTCCAGGAAGGAGAAGG-3′) were used to genotype the mice (WT = 439 bp, Mutant = 401 bp). PCR products were sequenced on a 3,130xl Genetic Analyzer (Applied Biosystems). The *Lrfn2^−/−^* allele was backcrossed onto C57Bl/6J mice for 10 generations. The mutant allele has a 38 bp deletion in exon 2 of the *Lrfn2* gene (chr17:49,070,008–49,070,045 GRCm38/mm10) causing a frameshift mutation that is predicted to be a null allele.

The *Elfn2^−/−^* mouse was created by JAX labs using CRISPR/Cas9 technology and has a 7,493 bp deletion (chr15:78667284–78674776; GRCm38/mm10) that removes the entire open reading frame for *Elfn2*, which is encoded by a single exon. To genotype the *Elfn2* line, PCR as described above using primers Elfn2-F: 5′-AGACAGTCCCTACCCACACG-3′; ELFN2-WTR:5′-AGGCTCAGACCTTCAAGCAG-3′; ELFN2-KOR:5′- ACCAGGTTGTCAGCACATCA-3′, yields a 321 bp fragment for the WT allele, and 301 bp fragment for the deleted allele.

### Antibodies

Antibodies used in this study are listed in Table 1. The goat anti-mGluR6 antibody was generated commercially (Life Technology Corporation) by immunizing animals with the peptide KKTSTMAAPPKSENSEDAK conjugated to KLH, and affinity purified using the peptide conjugated to a solid support. The specificity of all antibodies were validated on retinal sections from the respective knock-out mouse lines.

**Table 1.** Immunohistochemical reagents used in experiments

Antigen	Dilution	Source	Catalogue #
LRFN2	1:1,000	Sigma	SAB3500015
LRIT3	1:1,000	Hasan et al. (2019)	N/A
GPR179	1:2,000	Peachey et al. (2012)	N/A
TRPM1	1:1,000	Hasan et al. (2019)	N/A
mGluR6	1:1,000	Current paper	N/A
Pikachurin	1:2,000	Wako Chemicals	011-22631;
PNA	1:1,000	Molecular probes	L-32460;
ELFN2	1:2,000	Invitrogen	PA5-43521,

#### Retinal preparation and immunohistochemistry

This was done as previously described (Pearring et al., 2011; Hasan et al., 2016). Mice were killed, their eyes were enucleated, and the lens removed. Eyecups were fixed for 15–30 min in 4% paraformaldehyde in phosphate buffer (0.1 M PB), pH 7.4. After fixing, the eyecups were washed three times in PBS then cryoprotected in increasing concentrations of sucrose in PB (5%, 10%, 15% for 1 h each, and 20% overnight). Eyecups, usually from multiple genotypes, were embedded in 2:1 OCT/20% sucrose solution and frozen in a liquid nitrogen-cooled bath of isopentane. Eyecups were sectioned (18 µm) using a Leica 1,850 cryostat, mounted on Superfrost Plus glass slides (Thermo Fisher Scientific), and stored at −80°C. Before immunostaining, sections were warmed to 37°C and washed with PBS for 5 min and PBS containing 0.5% Triton X-100 (PBX) for 5 min. After blocking in PBX containing 5% normal donkey serum (blocking solution) for 1 h, sections were incubated with primary antibody diluted in blocking solution overnight at room temperature, and then washed three times for 10 min each with PBX. Sections were incubated with secondary antibody (1:1,000) in PBX for 1 h at room temperature followed by washing for 10 min twice in PBX and once in PBS. Coverslips were mounted to slides using Vectashield (Vector Laboratories). Images were taken using an FV3000 confocal microscope (Olympus) and corrected for contrast and brightness using FluoView Software (Olympus) or Photoshop (Adobe Systems). Representative images are shown in the figures (typically four sections, each containing multiple genotypes, were placed on a single slide). Retinas from at least three mice were processed and examined.

### Mass spectrometry

Mice were killed by CO_2_ exposure, retinas were isolated from control mice and homogenized in lysis buffer (1% Nonidet P-40, 2 mm EDTA (ethylenediaminetetraacetic acid), and 20 mm HEPES (4-(2-hydroxyethyl)-1-piperazineethanesulfonic acid), pH 7.4, supplemented with protease inhibitor cocktail (Sigma) by rotating for 45 min at 4°C. Samples were centrifuged at 17,000 × *g* for 20 min at 4°C to remove the cell debris, and supernatant was pre-cleared with Dynabeads (Invitrogen) for 1 h at 4°C. Samples were incubated with anti-LRFN2 or anti-TRPM1 antibodies overnight at 4°C. Dynabeads were added to lysates and incubated for 1.5 h at 4°C. Protein complexes were eluted from Dynabeads with NuPAGE LDS sample buffer (Invitrogen) and electrophoresed on NuPAGE gels (Invitrogen), until the highest molecular weight standard (260 kDa) had moved ∼5 mm into the gel. Electrophoresed gel pieces were cut from the top of the gel and an in-gel tryptic digestion was performed.

The resulting peptide mixture was resolved by liquid chromatography (LC) using an EASY n-LC (Thermo Scientific) UHPLC system with buffer A (2% v/v acetonitrile/0.1% v/v formic acid) and buffer B (80% v/v acetonitrile/0.1% v/v formic acid) as mobile phases. The mass spectrometry data from LC elutes was collected using an Orbitrap Elite ETD mass spectrometer (Thermo Scientific). A decision tree was used to determine whether CID or ETD activation was used. Proteome Discoverer v1.3.0.330 was used to analyze the data collected by the mass spectrometer. Scaffold software (version 4.10.0) was used to calculate the false discovery rate using the peptide and protein prophet algorithms.

### In situ hybridization

We used the probes and in situ hybridization kits as recommended by the manufacture, (ACD a Bio-Techne). The only deviation from their protocol was that we reduced the denaturation step of boiling the sections on slides from 10 min to 1 min to prevent loss of sections.

### Electroretinography (ERG)

ERGs were obtained as described previously (Peachey et al., 2012; Kinoshita and Peachey, 2018). Mice were dark-adapted overnight and anesthetized with ketamine (117.5 mg/kg) and xylazine (11.3 mg/kg) solution prepared in Ringer's solution, and prepared for ERG recordings using dim red light. Pupils were dilated with topical applications of 0.625% phenylephrine hydrochloride and 0.25% Tropicamide and the corneal surface anesthetized using 1% proparacaine HCL. Body temperature was maintained via an electric heating pad (TC1000 Temperature control, CWE Inc.). ERGs were recorded using a clear contact lens with a gold electrode contacting the corneal surface wetted with 1% methylcellulose. Needle electrodes in the tail and on the midline of the forehead serve as a ground and reference, respectively. Scotopic responses were recorded by presenting light flashes (from −3.6 to 1.4 log cd sec/m^2^) to dark adapted mice. For photopic ERGs, mice were light adapted to 20 cd/m^2^ for 5 min and responses were measured by presenting light flashes (from −0.8 to 1.4 log cd sec/m^2^) on this rod saturating background.

### Statistical analyses

Prism 10.1.2 software (GraphPad Software, Inc.) was used to perform the appropriate statistical analyses (see text and figure legends) for the necessary comparison. Statistical significance was determined at *p* ≤ 0.05.

## Results

**Figure 1.**
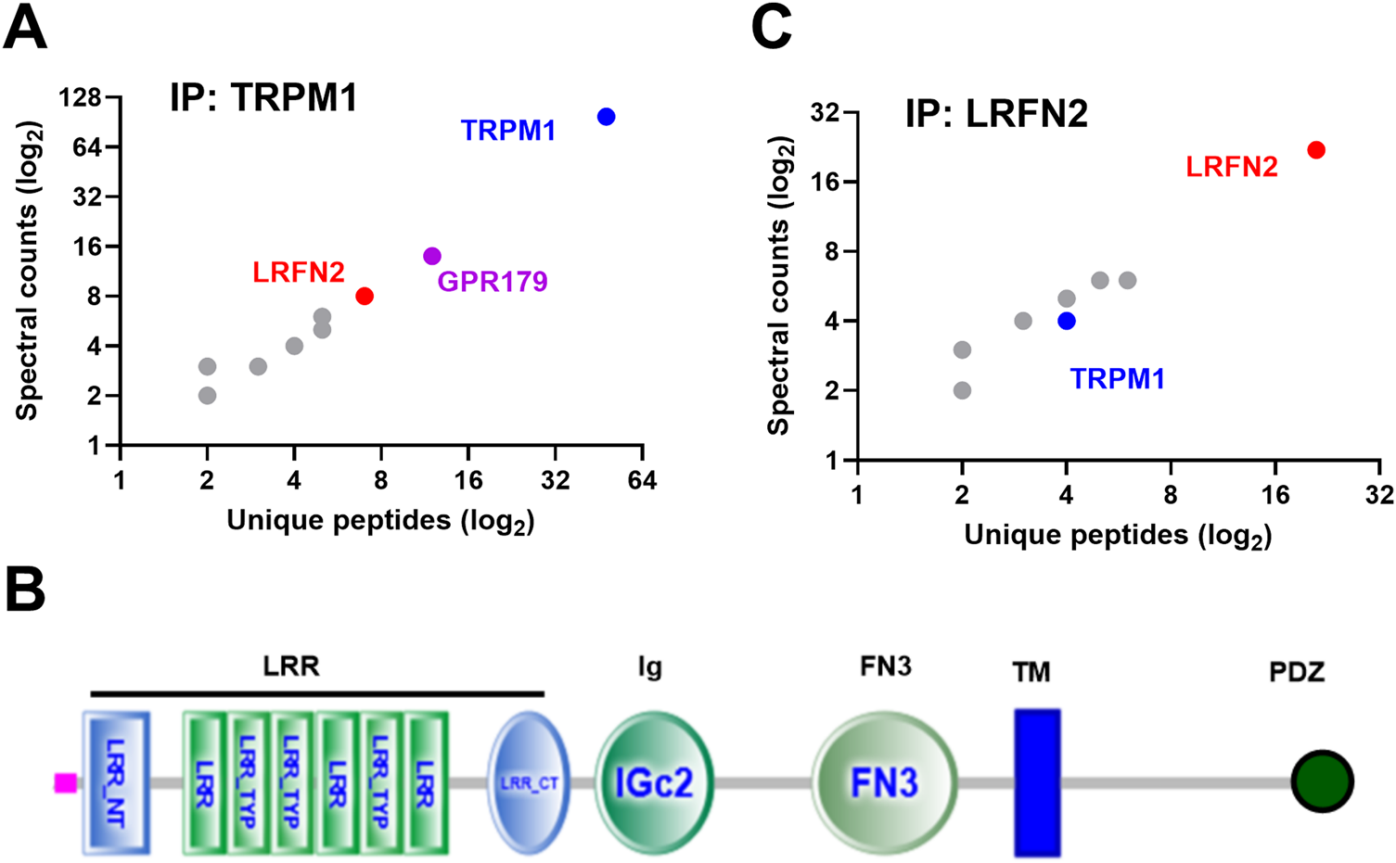
LRFN2 is located at the DBC signalplex in mouse retina. ***A***, Mass spectrometry analysis of proteins copurified with antibodies to TRPM1. ***B***, Schematic diagram of the domain structure of LRFN2 predicted by the SMART® program. LRFN2 contains a signal sequence (purple box), extracellular LRR, Ig and FN3 domains, a transmembrane segment (TM, blue box), and intracellular C terminus containing a PDZ binding domain (green ball). ***C***, Mass spectrometry analysis of proteins copurified with antibodies to LRFN2. For full list of proteins see Mendeley Data, V1, doi: 10.17632/wphby2csr5.1.

TRPM1 is a non-specific cation channel that is part of a signalplex that includes mGluR6 and GPR179 and is critical to the function of DBCs. To identify additional signalplex proteins, we designed a proteomic screen, using TRPM1-specific antibodies to immunoprecipitate the TRPM1 complex from protein lysates prepared from control mouse retinas. As a control, we used a non-immune IgG to immunoprecipitate proteins from control retina lysates. Mass spectrometry of the immunoprecipitates resulted in the identification of thousands of peptides that could be mapped to hundreds of proteins. Analysis of proteins immunoprecipitated specifically with the TRPM1 antibody revealed the identification of a known interacting partner, GPR179 (Ray et al., 2014), in addition to TRPM1 itself (48 unique peptides; 37% coverage) (Fig. 1*A*). We then filtered the results for cell adhesion molecules and identified LRFN2, which was identified by seven unique peptides, with coverage of 13% of the amino acid sequence (Fig. 1*A*). LRFN2 is a type 1 single pass transmembrane protein with 3 predicted extracellular domains (Fig. 1*B*). To validate the TRPM1 immunoprecipitation result we used antibodies to LRFN2 in immunoprecipitation experiments, followed by mass spectrometry. This experiment identified 21 unique LRFN2 peptides (36% coverage) and 4 unique peptides that matched TRPM1 (3.4% sequence coverage, Fig. 1*C*). No TRPM1 or LRFN2 peptides were identified from IgG controls. For the full list of proteins identified see Mendeley Data, V1, doi: 10.17632/wphby2csr5.1. Based on these data we hypothesize that TRPM1 and LRFN2 are part of the same signaling complex. We also attempted to identify LRFN2 directly in immunoprecipitates (IP) using TRPM1 antibodies on western blots, and vice versa but were unable to detect LRFN2 in the IP product. We believe this is likely a sensitivity issue because LRFN2 is only expressed on cones, compared to TRPM1 which is on all DBCs. This results in levels of LRFN2 below our detection limit on western blots. Even when staining for LRFN2 in control retinal lysates the staining intensity of LRFN2 is low compared to other signalplex components. This was not a limitation for identification of LRFN2 in immunoprecipitates using mass spectrometry experiments.

### LRFN2 is expressed on cone pedicles in mouse retina

**Figure 2.**
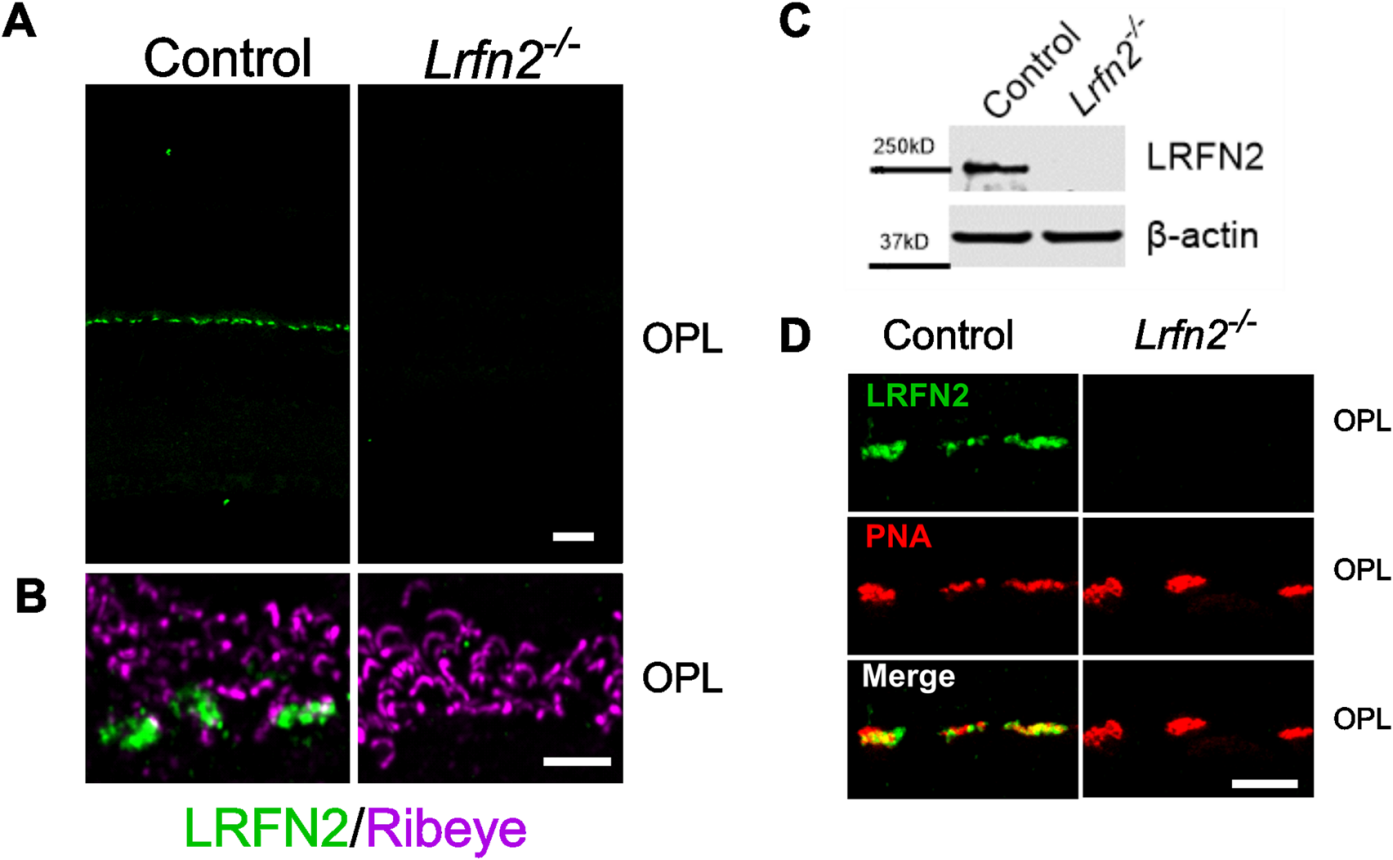
LRFN2 is localized to cone synapses in the OPL of mouse retina. ***A***, Representative confocal images after immunohistochemical staining for LRFN2 (green) in retina slices from control (left panels) and *Lrfn2^−/−^* retinas (right panels). Scale bar *A* = 5 µm; ***B,*** Representative confocal images of the OPL after immunohistochemical staining for LRFN2 (green) and Ribeye (magenta) in retina slices from control (left panels) and *Lrfn2^−/−^* retinas (right panels). Scale bar = 20 µm. ***C***, Western blot showing presence of LRFN2 in control retinal lysates, and its absence in *Lrfn2^−/−^* retinal lysates. β-actin was used as a loading control. 100 µg protein from retinal lysates was loaded/lane. ***D***, Immunohistochemical localization of LRFN2 (green) with the cone terminal maker PNA (red) in retinal sections from control and *Lrfn2^−/−^* retinas. Scale bar = 5 µm.

To determine the function of LRFN2 in retina and validate antibodies, we generated a *Lrfn2^−/−^* mouse using zinc finger nucleases. Several lines containing indels were created and we characterized one that had a 38 base pair deletion within exon 2. This created a frameshift mutation and was expected to be a null allele. To determine the expression pattern of LRFN2 in retina we used a commercially available antibody (Sigma, Cat# SAB3500015). To verify its specificity we compared staining of control and *Lrfn2*^−*/*−^ retinas, using both immunohistochemistry and western blotting (Fig. 2). The LRFN2 antibody showed staining exclusively in the OPL of the control retina, and was absent in the *Lrfn2*^−*/*−^ retina (Fig. 2*A,B*). Western blots also showed that the antibody was specific to LRFN2, staining a 250 kd band in control, which was absent in the *Lrfn2^−/−^* retina lysates (Fig. 2*C*).

The staining pattern in the control retina OPL (Fig. 2*B*) resembles that seen for the cone terminal marker PNA (Blanks and Johnson, 1983). To evaluate this we double labelled transverse retinal sections for LRFN2 and for PNA. Figure 2*D* shows that LRFN2 and PNA colocalize. Because of the imaging resolution it is not possible to determine if LRFN2 is expressed in cones or postsynaptic cone BCs or HCs.

**Figure 3.**
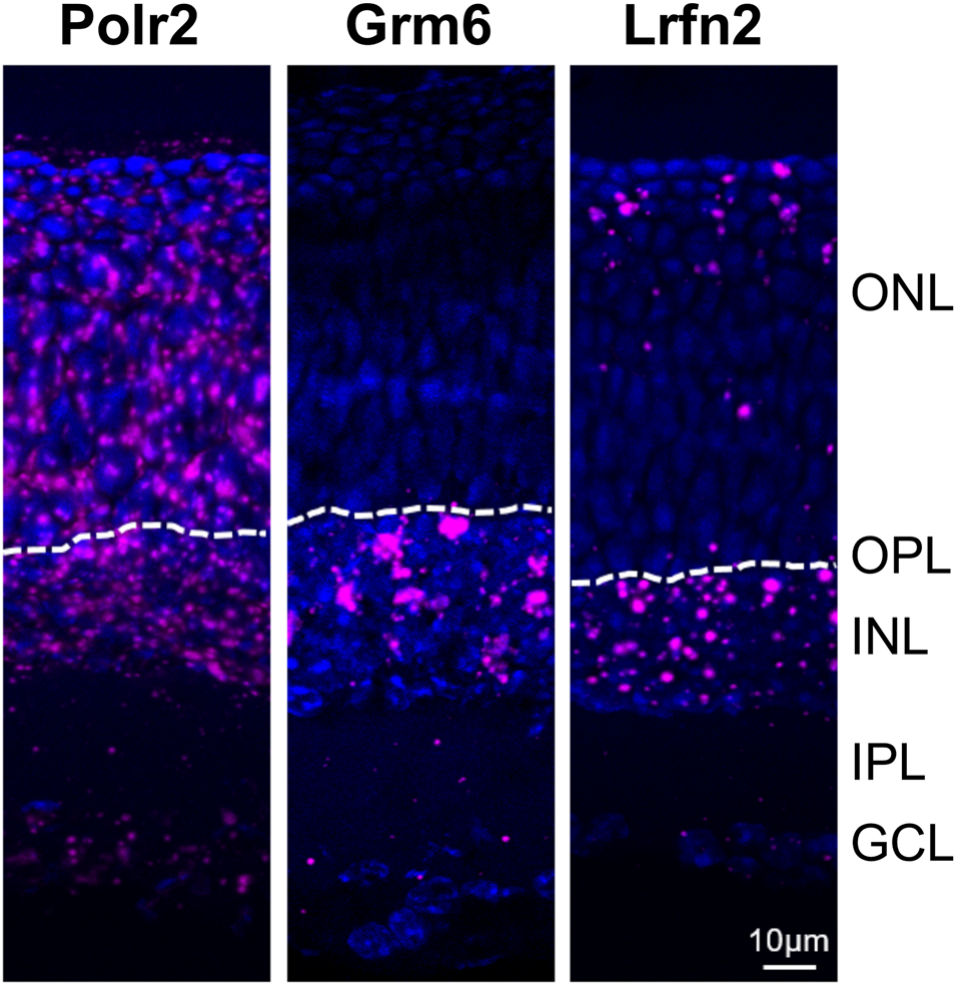
*Lrfn2* mRNA is expressed in the ONL and INL. In situ hybridization of mRNAs from *Polr2* (RNA polymerase II as a positive control), *Grm6* as a representative DBC signalplex member, and *Lrfn2*. The dashed lines shows the location of the OPL (outer plexiform layer), ONL, outer nuclear layer; INL, inner nuclear layer; IPL, inner plexiform layer; GCL, ganglion cell layer. Dot plots from scRNAseq are shown in Extended Data Figure 3-1.

To examine the cell types expressing LRFN2 we used in situ hybridization for *Lrfn2* mRNA using RNAscope (Fig. 3). We used probes to RNA polymerase 2 (Polr2) mRNA as a positive control to mark all cells, and probes to mRNA from *Grm6* to mark DBCs. *Polr2* shows signal in all cell layers in the retina and *Grm6* shows staining limited to the INL. The data for *Lrfn2* show it is present in a cell population at the outer region of the ONL, consistent with the location of cone cell bodies (Rich et al., 1997). The in situ data also shows expression of *Lrfn2* in a cell population in the INL, which could be a subset of bipolar and/or amacrine cells. To address this question further we examined publically available single cell mRNA sequencing data from human (Wang et al., 2022) and P14 mouse (Macosko et al., 2015) retina (Extended Data Fig. 3-1). LRFN2 expression is enriched in cones of both species, consistent with the in situ data. Further, examination of data from P14 mouse retina (Macosko et al., 2015), mouse bipolar (Shekhar et al., 2016) or amacrine (Yan et al., 2020) cell datasets failed to identify a cell type that might represent those in the INL expressing *Lrfn2* mRNA (see Extended Data Fig. 3-1). What these data do reveal is that the expression level of *Lrfn2* is quite low in these cells compared to say *Grm6* mRNAs levels in DBCs. Thus the identity of the *Lrfn2* mRNA expressing cell population in the INL remains to be defined.

10.1523/ENEURO.0120-23.2024.f3-1 Extended Data Figure 3-1 Single cell RNAseq data shows a relatively low level expression of Lrfn2 in retina cells. ***A.*** Dot plot for select genes in P14 mouse retina. Lrfn2 expression is highest in cones. Data set: C57B6 wild-type P14 retina by drop-seq (Macosko EZ, et al. (2015) Highly Parallel Genome-wide Expression Profiling of Individual Cells Using Nanoliter Droplets. Cell 161:1202-1214.) ***B,*** Dotplot of Lrfn2 and select gene mRNA expression in retinal bipolar cells. Data set: Retinal Bipolar Neuron Drop-seq Shekhar K et al. (2016) Comprehensive Classification of Retinal Bipolar Neurons by Single-Cell Transcriptomics. Cell 166:1308-1323 e1330. ***C,*** Dotplot of Lrfn2 and select gene mRNA expression in retinal amacrine cells. All data are from the Single Cell Portal (https://singlecell.broadinstitute.org/single_cell). Data set: Mouse Retinal Cell Atlas: Molecular Identification of over Sixty Amacrine Cell Types (Yan W et al. (2020) Mouse Retinal Cell Atlas: Molecular Identification of over Sixty Amacrine Cell Types. J Neurosci 40:5177-5195.). Download Extended Data Figure 3-1, TIF file.

### ON bipolar cell signalplex protein expression in *Lrfn2*^−*/*−^ retina

**Figure 4.**
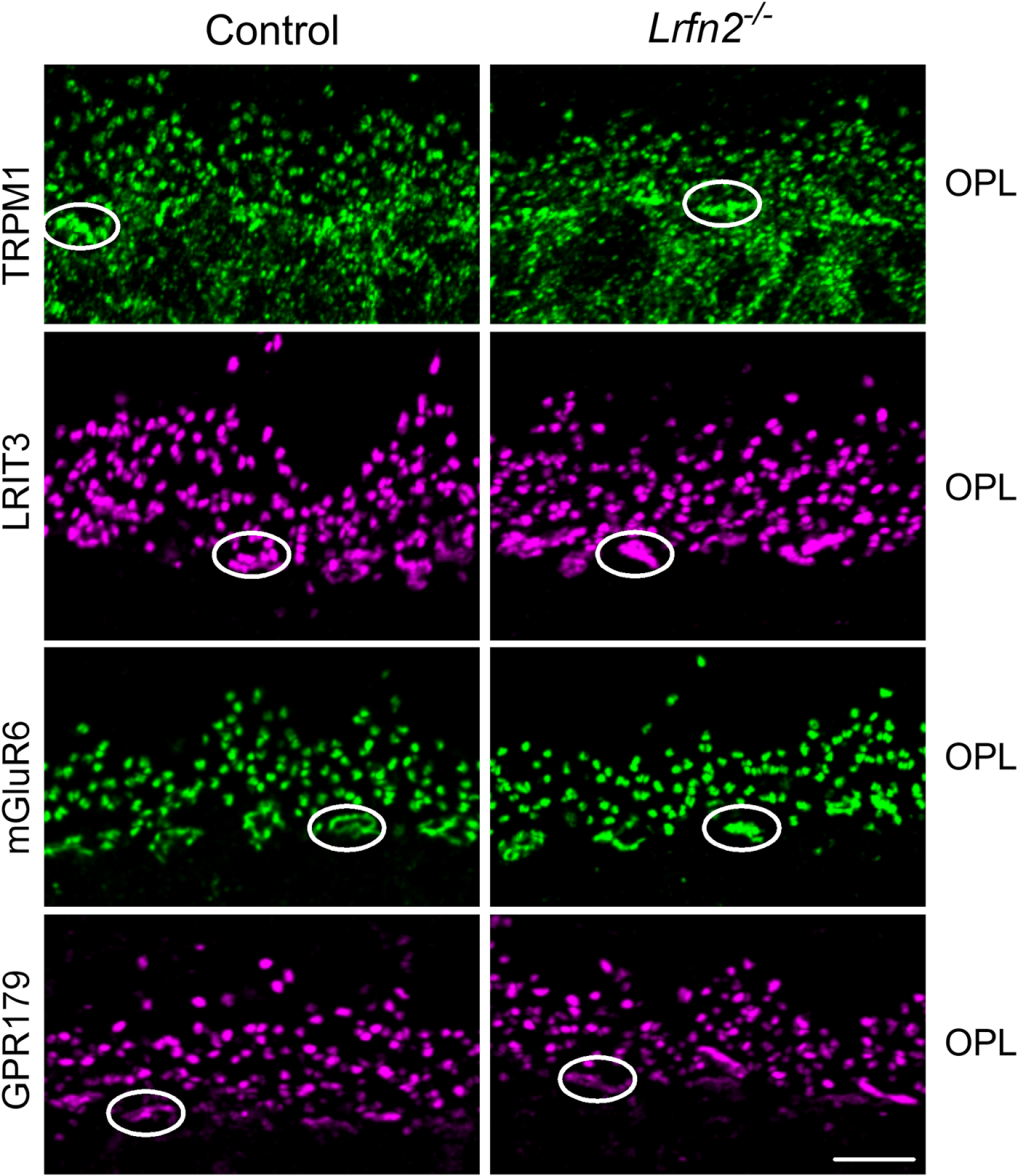
Synaptic proteins are localized normally in *Lrfn2^−/−^* mouse retina. Representative confocal images showing immunohistochemical localization of synaptic proteins TRPM1, LRIT3, mGluR6 and GPR179 in the OPL of transverse retinal slices from control and *Lrfn2^−/−^* retinas. Scale bar = 5 µm. Circles indicate cone synapses. OPL, outer plexiform layer. Validation of the mGluR6 antibody is shown in Extended Data Figure 4-1.

**Figure 5.**
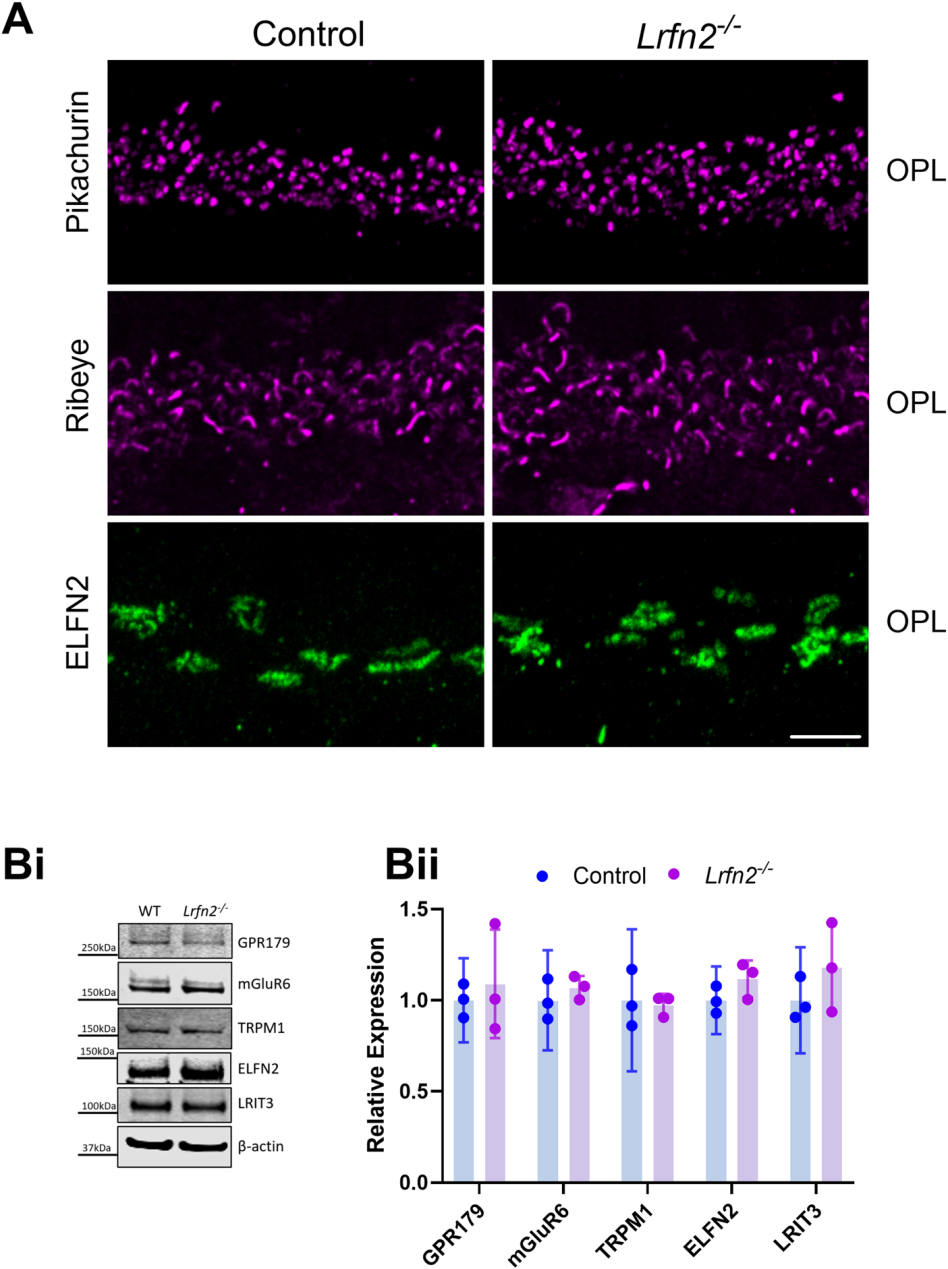
Synaptic proteins pikachurin, ribeye and ELFN2 are localized normally in absence of LRFN2. ***A***, Representative confocal images after immunohistochemistry for Pikachurin (top row), Ribeye (middle row), and ELFN2 (bottom row) in transverse retinal sections from control and *Lrfn2^−/−^* mouse retina. Scale bar = 5 µm. OPL, outer plexiform layer. ***Bi***, Representative western blots of retinal lysates for DBC signalplex proteins, ***Bii***, Quantification of signalplex protein levels from western blots of retinal lysates from 3 mice of each genotype (mean ± 95% CI). Data were normalized to the control sample. β-actin was used as a loading control.

Previous studies analyzing various knock-out mouse lines have shown that there is a complex relationship and interdependency between the expression of DBC signalplex proteins (Gregg et al., 2014). For example, nyctalopin is required for TRPM1 insertion at the DBC dendritic tips (Pearring et al., 2011). LRIT3 is required for localization of mGluR6, TRPM1, nyctalopin, GPR179, and RGS7/11 in cone DBCS (Neuille et al., 2015, 2017; Hasan et al., 2020). ELFN1 and ELFN2 are required for mGluR6 insertion into rod and cone DBCs, respectively (Cao et al., 2020, 2022). To examine if LRFN2 was required for and dependent on other DBC signalplex proteins we examined expression in knock-out mice. We used immunohistochemistry to examine the expression of TRPM1, LRIT3, mGluR6, GPR179, Pikachurin, Ribeye and ELFN2, which are expressed at cone to cone DBC synapses, in control and *Lrfn2^−/−^* retinas. In all cases the expression pattern in the *Lrfn2^−/−^* mice was indistinguishable from controls (Figs. 4, 5). To determine if there were any quantitative changes in expression of mGluR6, TRPM1, GPR179, ELFN2, and LRIT3 we analyzed data from western blots (Fig. 5*Bi*) and quantified the results from 3 different animals (Fig. 5*Bii*). Consistent with the immunohistochemical observations, these data show that there were no changes in expression level of the tested signalplex components (*p* > 0.05 for genotype; 2-way ANOVA, with post hoc adjustment for multiple testing).

10.1523/ENEURO.0120-23.2024.f4-1 Extended Data Figure 4-1 mGluR6 antibody is specific. We developed an antibody to mGluR6 as described in the methods section. Serum was collected and mGluR6 antibody affinity purified. ***A,*** The purified mGluR6 antibody (green) was used to stain Control and *Grm6^-/-^* retina sections. The only staining was as puncta at the OPL. ***B,*** High power images of staining with mGluR6 antibody (green) and the cone terminal marker PNA (magenta). The data demonstrate punctate staining in the outer plexiform layer (OPL). The merged image shows green puncta from rod to rod BC synapses, and the large magenta/white puncta staining is from cone terminals. Scale bar in A = 10µm and in B = 5µm. Download Extended Data Figure 4-1, TIF file.

**Figure 6.**
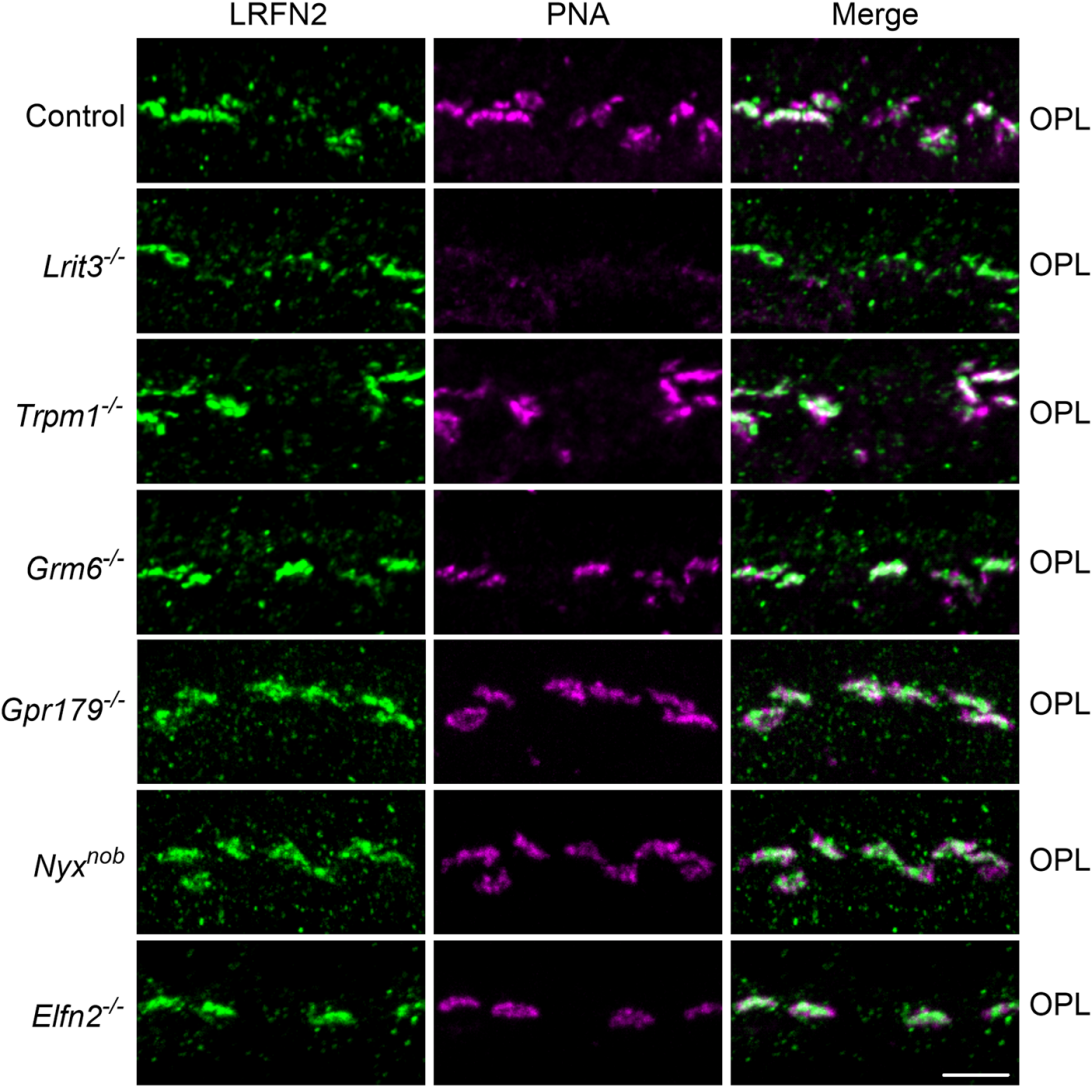
LRFN2 is expressed normally in control, *Lrit3^−/−^, Trpm1^−/−^*, *Grm6^−/−^*, *Gpr179^−/−^*, *Nyx^nob^* and *Elfn2^−/−^* retinas. Images are from representative of the OPL from retinal sections (*n* = 3) stained for LRFN2 (green) and PNA (magenta). Scale bar = 5 µm. OPL, outer plexiform layer.

To determine if the expression of LRFN2 was dependent on signalplex components LRIT3, TRPM1, mGluR6, GPR179, Nyctalopin, or ELFN2, we determined its expression pattern in control, *Lrit3*^−*/*−^*, Trpm1*^−*/*−^, *Grm6*^−*/*−^, *Gpr179^−/−^*, *Nyx^nob^*, and *Elfn2^−/−^* knock-out mouse lines, respectively (Fig. 6). To mark the cone terminals in each section we stained with PNA. In all these knock-out lines the staining pattern for LRFN2 was indistinguishable from controls. Combined these data showed that the expression of LRFN2 was not dependent on the expression of any post- (mGluR6, TRPM1, GPR179, Nyctalopin) or pre-synaptic (LRIT3 or ELFN2) protein tested. The low level of PNA staining in *Lrit3^−/−^* mice, has been reported previously (Neuille et al., 2015; Hasan et al., 2020), although the cause is currently unknown. Thus, LRFN2 localization to the cone synaptic terminal is not dependent on known signalplex members, nor is there any interdependency of LRFN2 with several other DBC signalplex members.

### Function of LRFN2 in signal transduction in mouse retina

**Figure 7.**
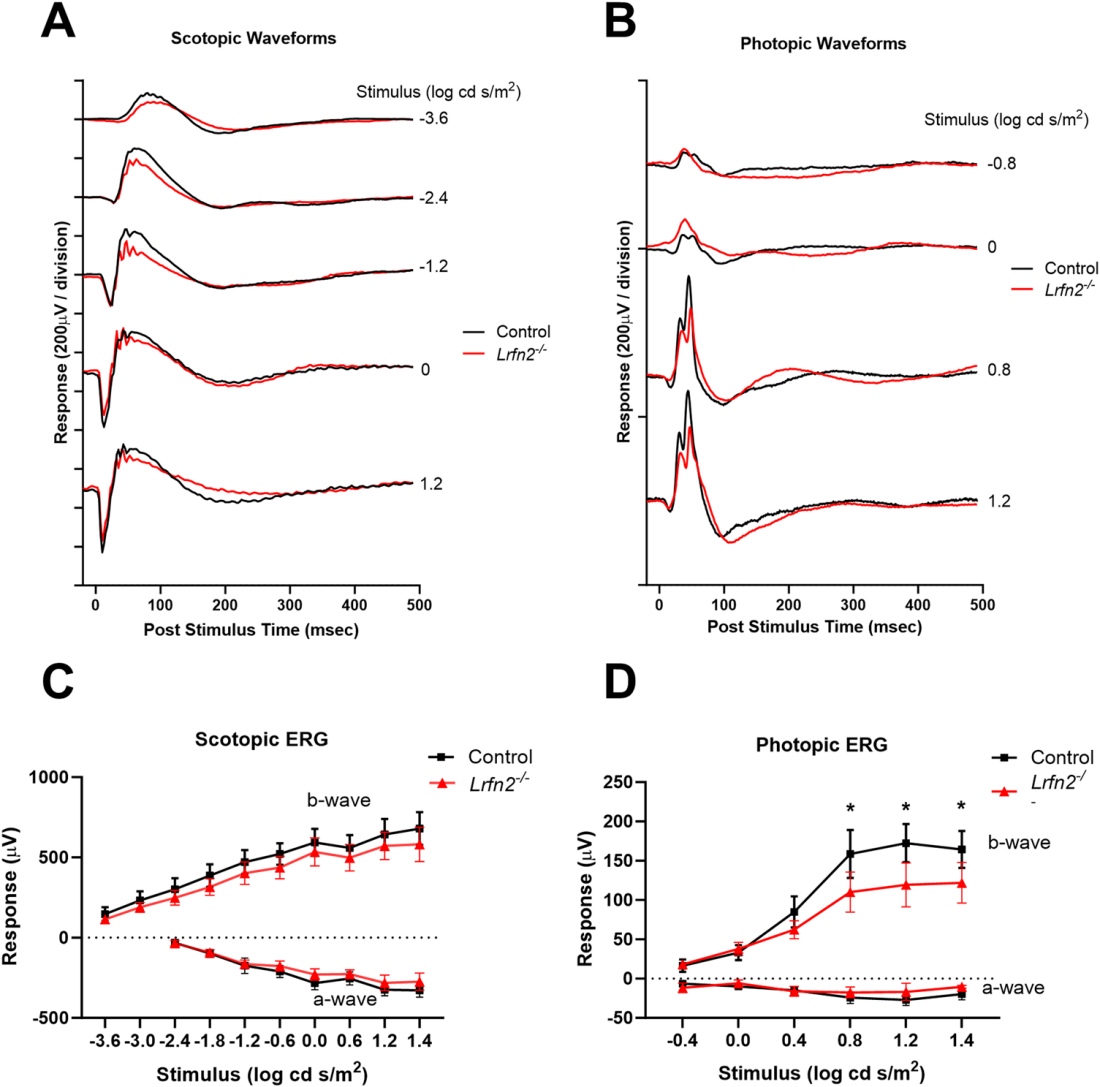
Photopic ERG b-waves of *Lrfn2^−/−^* mice have a reduced amplitude. Electroretinogram waveforms for a single animal under ***A,*** scotopic and ***B,*** photopic conditions at different intensity flashes for a control (black) and a *Lrfn2^−/−^* (red) eye. Average stimulus-response plots for the ERG a-wave and b-wave amplitudes under ***C,*** scotopic and ***D,*** photopic conditions for control (*n* = 7, black) and *Lrfn2^−/−^* (*n* = 8, red). Statistics: comparison of control vs *Lrfn2^−/−^* groups, *p_adj _< 0.05 for stimuli, 2-way repeated measures ANOVA. Between the groups, there are no differences in b-wave amplitude under scotopic conditions, and in a-wave amplitude under either scotopic or photopic conditions (2-way repeated measures ANOVA, *p*_adj _> 0.05). 95% confidence intervals of the differences between genotypes are shown in Extended Data Figure 7-1.

To assess the possible impact of loss of LRFN2 on function we used the electroretinogram (ERG), which assesses photoreceptor, and connected DBC function. Recordings were done at 6–8 weeks of age under either scotopic or photopic conditions, to assess rod BC and cone DBC function, respectively. The waveforms for several flash intensities in a control and *Lrfn2^−/−^* mouse are shown under scotopic (Fig. 7*A*) and photopic (Fig. 7*B*) conditions. Summary data for control (*n* = 7) and *Lrfn2^−/−^* (*n* = 8) are shown in Figure 7*C* and *D*, respectively. As expected given LRFN2 is not expressed at the rod to rod BC synapse, the a-wave or b-wave amplitudes of the electroretinogram under scotopic conditions in *Lrfn2^−/−^* mice were not different than controls (Fig. 7*A,C*). The photopic ERG, which reflects cone-pathway function, showed a normal a-wave at all flash intensities (Fig. 7*B,D*). In contrast, the b-wave amplitudes of the photopic ERG in *Lrfn2*^−*/*−^ mice were significantly reduced at the 3 highest flash intensities tested Figure 7*D*. (2-way ANOVA, adjusted for multiple testing using the Sidak correction). The estimation plots of the 95% CI of the difference between controls and *Lrfn2^−/−^* for b-waves, under scotopic and photopic conditions are shown in Extended Figure 7-1. This reduction in photopic b-wave amplitude in the *Lrfn2^−/−^* mice indicates an alteration in signal transduction between cones and cone DBCs.

10.1523/ENEURO.0120-23.2024.f7-1 Extended Data Figure 7-1 Confidence intervals of differences between Control and *Lrfn2^-/-^.* Data were analyzed using 2-way ANOVA, with post hoc comparisons adjusted using Šídák's multiple comparisons test for multiple testing adjustment. The only values showing a significant difference at Padj ≤ 0.05 are shown in red. Download Extended Data Figure 7-1, TIF file.

## Discussion

Here we show that LRFN2 is part of the cone DBC signalplex, and likely represents another member of the trans-synaptic proteins required for normal visual function. LRFN2 contains LRR, Ig and Fn3 domains predicted to be extracellular. The intracellular domain of ∼130 amino acids also contains a PDZ binding domain (Morimura et al., 2006). Cones make synapses with, and relay high-sensitivity signals to many subtypes of ON and OFF cone bipolar cells. Our proteomic approach identified LRFN2 as another novel DBC signalplex protein.

LRFN2 has been studied in the brain (Thevenon et al., 2016; Morimura et al., 2017; Li et al., 2018; McMillan et al., 2021), and is a presynaptic organizer of synapse development by promoting F-actin/PIP2-dependent clustering of Neurexin in hippocampal neurons (Brouwer et al., 2019). In *Lrfn2^−/−^* mice there is a decrease in synaptic plasticity and inhibitory synapse development (Li et al., 2018). These mice also have altered behaviors, specifically decreased acoustic vocalization of pups and increased startle response, although there was no effect on locomotion. A microdeletion in humans containing the *LRFN2* gene was found associated with selective working memory deficits, and borderline intellectual functioning (Thevenon et al., 2016).

LRFN2 associates with and regulates surface expression of AMPA receptors, synaptic activity and hippocampal long-term potentiation through interaction with nexin-27 (McMillan et al., 2021). Whether LRFN2 interacts with nexin's at the cone terminal is unknown. Our in situ hybridization data shows expression in both the ONL and INL. The single cell RNA sequencing data (Peng et al., 2019; Wang et al., 2022) suggest its expression is in cones in the outer retina. This is consistent with the fact that LRFN2 contains a PDZ binding domain (Morimura et al., 2006) that could interact with PSD95, which is a presynaptic protein in the retina (Koulen et al., 1998). Thus, LRFN2, like LRIT3 (Hasan et al., 2019) and ELFN1 (Cao et al., 2015) and ELFN2 (Cao et al., 2020) likely serves as trans-synaptic scaffold between photoreceptors and DBCs. Given the expression of *Lrfn2* mRNA is present in population of INL cells it may be localized on the dendritic tips of cone DBCs and/or HBCs, sub-serving different functions at each location including interacting with AMPA receptors in HBC dendrites.

At the functional level the loss of LRFN2 causes no change in the scotopic ERG, consistent with expression only at cone synapses. However, it is required for the normal signaling of cone DBCs at bright flash intensities. One question that arises is if LRFN2 is part of the DBC signalplex why is the phenotype so subtle. One possibility is that because the *Lrfn2^−/−^* mouse lacks LRFN2 during development that there is compensation by another LRFN family member (LRFN1, 3, 4, or 5). scRNA seq studies show gene expression of all five members in both mouse and human retina (Balasubramanian et al., 2021; Wang et al., 2022), although currently their localization and developmental expression pattern is unknown. Such compensation was recently shown to occur for another cone-specific trans-synaptic protein, ELFN2 (Cao et al., 2020), the loss which had no effect on the synaptic function as measured by the ERG. Subsequent elegant studies using conditional knock-out mice, showed this was because ELFN1 was used to compensate for the absence of ELFN2 during development (Cao et al., 2022). The knock-out of ELFN2 in adult mice showed a decrease in the photopic b-wave because of decreased expression of mGluR6, indicating expression of mGluR6 in cone DBCs was dependent on ELFN2. Whether a similar type of compensation occurs after loss of LRFN2 remains to be determined.

In conclusion, our studies have identified a new LRR containing protein, LRFN2, that in the retina is expressed at cone synaptic terminals, and impacts signalplex function. The expression of *Lrfn2* mRNA in cones and a population of cone DBCs and/or HBCs means it could interact with the TRPM1 trans-synaptically or be part of the postsynaptic DBC signalplex, or it may have multiple independent roles at the cone synapse. Thus, LRFN2 is a member of a large family of LRR-containing proteins that includes FLRTs, NGLs, Slitrks, LRRTMs and SALMs, which are expressed throughout the CNS, and many have been shown to impact retinal synaptic structure and function (Schroeder and de Wit, 2018). The detailed method of action of LRFN2 and the function of its various domains remains for future studies.

## Synthesis

Reviewing Editor: Eran Perlson, Tel Aviv University Decisions are customarily a result of the Reviewing Editor and the peer reviewers coming together and discussing their recommendations until a consensus is reached. When revisions are invited, a fact-based synthesis statement explaining their decision and outlining what is needed to prepare a revision will be listed below. The following reviewer(s) agreed to reveal their identity: Christophe Ribelayga, Kirill Martemyanov.

The authors report the identification of a novel molecule (LRFN2) expressed at the synapse between cones and cone bipolar cells (BCs) and characterized its functional role. LRFN2 was identified initially by mass-spec screen as a binding partner for effector channel TRPM1 that mediates depolarizing response of retina ON-bipolar cells upon changes in photoreceptor inputs. The authors provide evidence that TRPM1 and LRFN2 can co-immunoprecipitate upon cotransfection in HEK293 cells and that LRFN2 is associated with cone synapses co-localizing with the cone-specific marker PNA at these sites. They further generated a mouse knockout of LRFN2 which shows no changes in the synaptic content of proteins found at the cone synapses. Conversely, no changes in LRFN2 at cone synapses were detected upon the knockout of other synaptic molecules. Intriguingly, despite any discernable molecular changes, LRFN2 knockout mice show reduced photopic b-wave possibly suggesting deficits in cone synaptic transmission.

Overall, this exciting paper reports on the discovery of LRFN2, a novel player of the cone-to-cone bipolar cell signaling complex. Still, the presented report is still preliminary with some gaps and deficiencies which make the claims poorly supported and the story incomplete.

1. TRPM1-LRFN2 interaction needs to be confirmed in native retinas. The authors clearly have both TRPM1 and LRFN1 antibodies. Please examine co-immunoprecipitation of TRPM1 and LRFN2 by Western and use TRPM1 KO retinas as a control in these experiments. Their antibodies clearly work for Western and knockouts are in hand.

2. Interactions between transmembrane proteins upon co-expression in HEK cells could be notoriously non-specific due to sticky nature of the TM regions and crowding effect on the cellular membranes. In fact, even with this setup the evidence of TRPM1 co-IP with LRFN2 is entirely lacking as figure 1F shows a blank blot. To establish interaction specificity in this heterologous system, especially given the trans-cellular nature as claimed, the authors should show that the proteins can bind when expressed in different cells or use soluble ectodomain of LRFN2 to bind to TRPM1 expressed again in separate cells.

3. The value of showing LRFN2 localization upon expression in HEK cells (Fig. 1D) is very limited. Please examine co-localization with TRPM1 in this experiment.

4. Showing data from public databases as an evidence for cell specific expression of LRFN2 is insufficient (Fig. 2D). It is a great starting point, but cellular specificity of expression needs to be established experimentally. It is routinely done by in situ hybridization.

5. It will be very helpful to see the expression of a few other proteins, e.g. synaptic markers and especially TRPM1 on the Western blot presented in Fig. 2B.

6. Data presented in Figs. 3, 4 and 5 would benefit from quantitative analysis. The changes in b-wave by ERG are rather small and could be driven by small changes in synaptic content of proteins.

7. ERG analysis is insufficiently developed. The photopic b-wave is studied on rod suppressing light background. Thus, the phenotype in the suppression of b-wave could be related to background adaptation rather than deficits in transmission per se. To disentangle this, one would have to perform ERG on dark adapted mice throughout scotopic and photopic intensities and extract the cone component of the response and then compare it with the light background data. Also, it will be quite helpful to quantify and present the b-wave amplitude data showing individual data points to appreciate variability as representative traces show very marginal difference between WT and KO mice in terms of the photopic b-waves.

8. Finally, the authors treatment of the literature when creating motivational narrative is somewhat selectively engineered and biased. It is a very small and focused field and the authors are surely intimately familiar with its history and major developments. Hence, it is disappointing to see treatment of foundational, as well as more recent work, on trans-synaptic interactions at photoreceptor synapses much of which is either ignored or inappropriately cited as an afterthought.

Some minor suggestions:

- Abstract (and other places in the text, including the significance statement): I encourage the authors to clarify whether they are referring to mice or humans when they state that there are 8 types of DBCs and 5 of HBCs. They should be more specific.

- Introduction: the 2/3 first sentences are confusing (e.g. convert photons into a glutamate signal...). This can be easily fixed.

At the bottom of the first page of Introduction, I encourage the authors to write a short paragraph on what is known of ELFNs. Also, more references are needed here.

- Methods: some of these techniques have been published already, please add refs.

- Fig. 1F: where is the TRPM1 signal (IP: LRFN2)?

- Discussion: I would start the first paragraph with the 4th sentence "Cones make..." and the following sentences.

- Extended figures are indicated in the text but not provided.

- Table 1: For mGluR6-Ab. I would like to see some characterization of this novel antibody in an extended figure.

- Figure 5: why is PNA labeling so weak in Lrit3-/- retina?

- Interaction between LRFN2 and TRPM1 in DBCs does not eliminate the possibility of LRFN2 interaction with AMPARs in HBCs. The authors should comment on that.
