# Supplementary figures and images for: Cone Synaptic Function is Modulated by the Leucine-Rich Repeat Adhesion Molecule LRFN2
Source: eNeuro. 2024 Mar 12;11(3):ENEURO.0120-23.2024. doi: 10.1523/ENEURO.0120-23.2024 (PMC10957230; doi:10.1523/ENEURO.0120-23.2024)

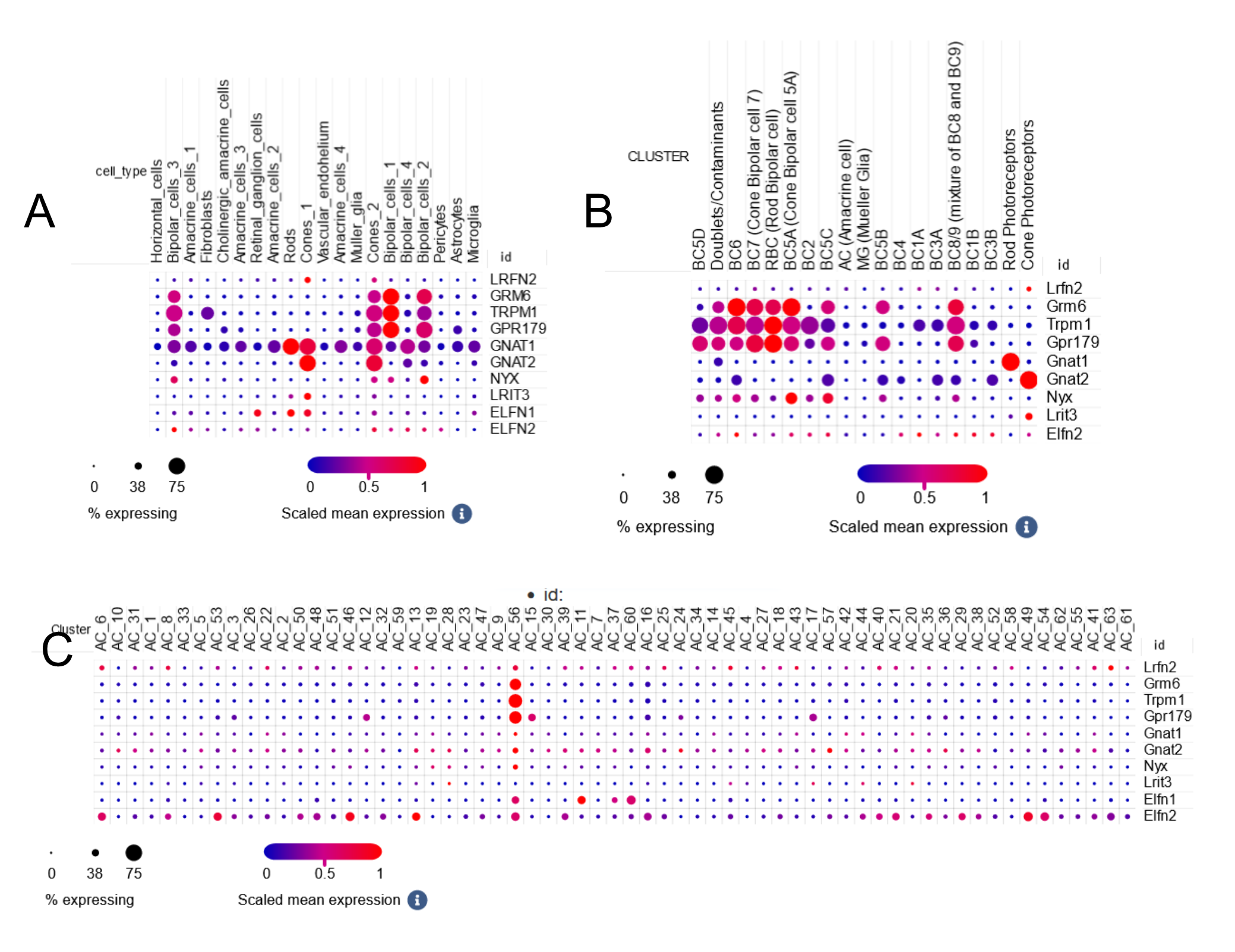

Supplement: Extended Data Figure 3-1 — Single cell RNAseq data shows a relatively low level expression of Lrfn2 in retina cells. A. Dot plot for select genes in P14 mouse retina. Lrfn2 expression is highest in cones. Data set: C57B6 wild-type P14 retina by drop-seq (Macosko EZ, et al. (2015) Highly Parallel Genome-wide Expression Profiling of Individual Cells Using Nanoliter Droplets. Cell 161:1202-1214.) B, Dotplot of Lrfn2 and select gene mRNA expression in retinal bipolar cells. Data set: Retinal Bipolar Neuron Drop-seq Shekhar K et al. (2016) Comprehensive Classification of Retinal Bipolar Neurons by Single-Cell Transcriptomics. Cell 166:1308-1323 e1330. C, Dotplot of Lrfn2 and select gene mRNA expression in retinal amacrine cells. All data are from the Single Cell Portal (https://singlecell.broadinstitute.org/single_cell). Data set: Mouse Retinal Cell Atlas: Molecular Identification of over Sixty Amacrine Cell Types (Yan W et al. (2020) Mouse Retinal Cell Atlas: Molecular Identification of over Sixty Amacrine Cell Types. J Neurosci 40:5177-5195.). Download Extended Data Figure 3-1, TIF file. [file eneuro-11-ENEURO.0120-23.2024-s001.tif]

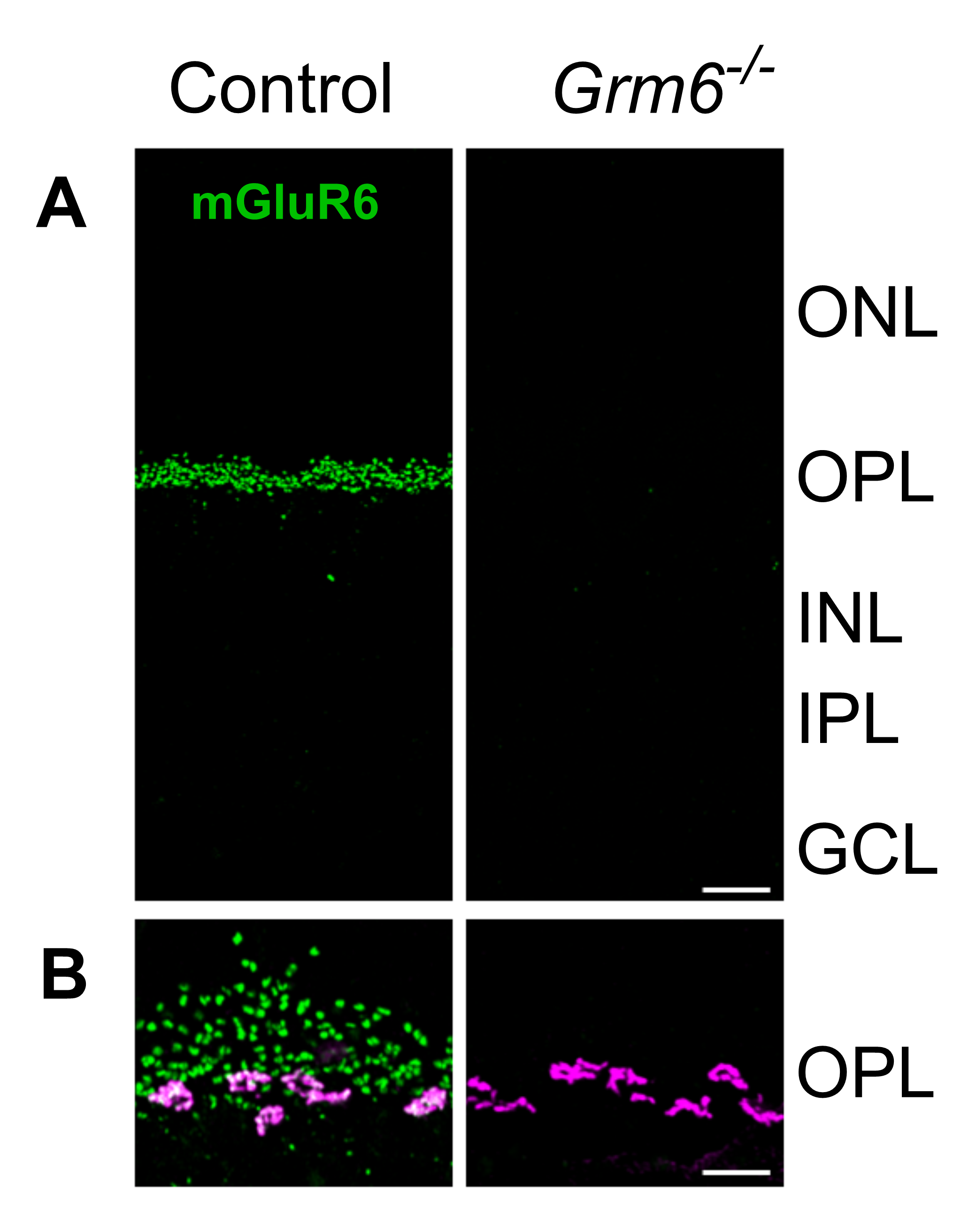

Supplement: Extended Data Figure 4-1 — mGluR6 antibody is specific. We developed an antibody to mGluR6 as described in the methods section. Serum was collected and mGluR6 antibody affinity purified. A, The purified mGluR6 antibody (green) was used to stain Control and Grm6-/- retina sections. The only staining was as puncta at the OPL. B, High power images of staining with mGluR6 antibody (green) and the cone terminal marker PNA (magenta). The data demonstrate punctate staining in the outer plexiform layer (OPL). The merged image shows green puncta from rod to rod BC synapses, and the large magenta/white puncta staining is from cone terminals. Scale bar in A = 10µm and in B = 5µm. Download Extended Data Figure 4-1, TIF file. [file eneuro-11-ENEURO.0120-23.2024-s002.tif]

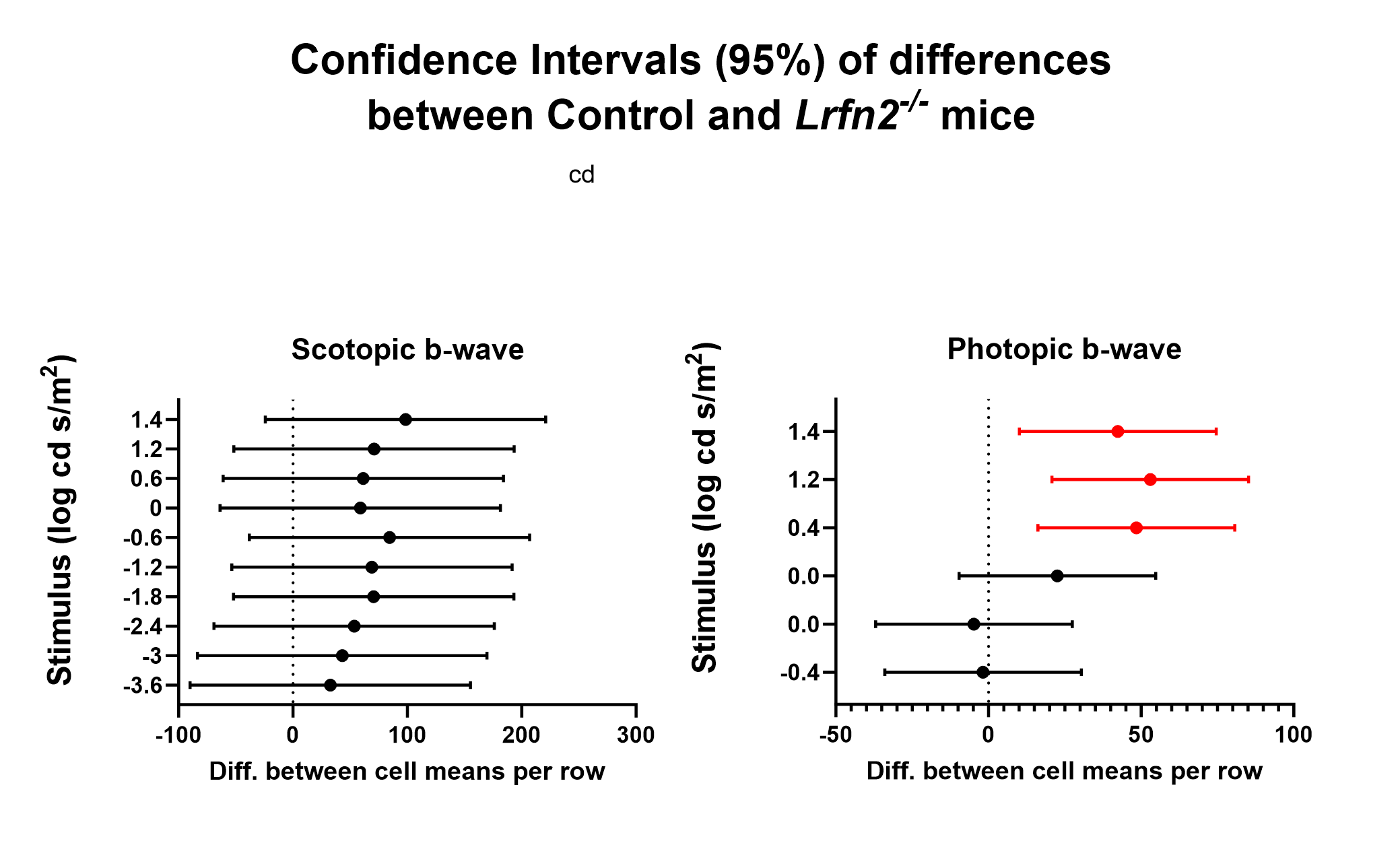

Supplement: Extended Data Figure 7-1 — Confidence intervals of differences between Control and Lrfn2-/-. Data were analyzed using 2-way ANOVA, with post hoc comparisons adjusted using Šídák's multiple comparisons test for multiple testing adjustment. The only values showing a significant difference at Padj ≤ 0.05 are shown in red. Download Extended Data Figure 7-1, TIF file. [file eneuro-11-ENEURO.0120-23.2024-s003.tif]
